# Cocoaine as an Anasthetic

**Published:** 1886-03

**Authors:** 


**Cocoaine as an Anasthetic in Fracture.**—Dr. J. R. Conway, Jr., reports in the *Medical Record* a case of fracture of the lower end of the radius reduced without pain after injection of cocoaine. He says: "All attempts at examination of the fracture caused great agony, and I resolved to try if deep injections of cocoaine at the point of fracture would sufficiently anesthetize the parts to allow of thorough examination and reduction of the deformity without causing pain. I proceeded to inject five minims of the four-per-cent. solution into the inner, outer, and posterior surfaces of the forearm directly over the seat of fracture and as deep as the bone. In five minutes the fracture could be thoroughly examined and even roughly handled without the patient experiencing the slightest pain. After the examination I reduced the deformity by extreme extension of the wrist joint together with traction, using considerable force, but without causing the patient any uncomfortable sensations.

